# Twin Reversed Arterial Perfusion

**DOI:** 10.7759/cureus.31116

**Published:** 2022-11-05

**Authors:** Punit Hans

**Affiliations:** 1 Obstetrics and Gynaecology, Patna Medical College, Patna, IND

**Keywords:** acardiac twin, twin pregnancy, monochorionic diamniotic, monochorionic, trap

## Abstract

Twin reversed arterial perfusion (TRAP) sequence is a rare condition found only in monochorionic pregnancies. It is a sequence in which a severely anomalous twin with an absent or rudimentary heart, also called an “acardiac twin,” is perfused by its co-twin. The cardiac system of this co-twin (pump twin) provides circulatory support to the acardiac twin from as early as the first trimester. The exact pathogenesis of TRAP sequence is still unknown. However, the mere presence of vascular anastomoses in the placenta alone cannot lead to the development of TRAP sequence. Moreover, the presence of retrograde blood flow through arterio-arterial anastomoses increases the possibility of TRAP sequence diagnosis in a suspected monochorionic twin pregnancy.

This report presents a case of TRAP sequence, which was undiagnosed till the delivery of twins. A 26-year-old patient gravida 3 para 2 (G3P2+0) with previous two normal deliveries, was referred to our emergency labor room at (assumed) 37 weeks of pregnancy, from a primary health center with complaints of vaginal leakage and a non-reassuring fetal status. She was immediately shifted to Operation Theatre and an emergency lower-segment cesarean section was performed. At the time of delivery, she was diagnosed as having a monochorionic diamniotic twin with TRAP sequence as one of the twins was acardiac, while the other was a live male baby (the pump twin) with massive ascites. A TRAP sequence pregnancy with a poor prognostic factor requires twin cord occlusion therapy or another form of intervention. Termination of the pregnancy is also an option, as it saves the patient from unnecessary obstetrical complications and morbidity.

## Introduction

Twin reversed arterial perfusion (TRAP) sequence is a rare condition found only in monochorionic pregnancies. It is a sequence in which a severely anomalous twin with an absent or rudimentary heart, also called an “acardiac twin,” is perfused by its co-twin. The co-twin that perfuses the acardiac twin is called the “pump twin.” This perfusion occurs via aberrant arterio-arterial anastomoses. Typically, the upper body and the head of the acardiac twin are poorly developed or absent altogether. Acardiac twins cannot survive outside the uterus. The extra circulatory burden on the pump twin as a result of providing perfusion to the acardiac twin puts it at risk of developing high-output cardiac failure. The complications arising from this may lead to a premature birth or even death. Among monochorionic twin pregnancies, the estimated incidence of TRAP sequence is 2.6% [1].

Comprehending normal fetal circulation can help in understanding the pathophysiology of TRAP sequence. Usually, fetal circulation involves the umbilical vein carrying the relatively oxygen-rich blood from the placenta to the fetus. Nearly 80% of placental blood is shunted into the inferior vena cava through the ductus venosus. The inferior vena cava carries the placental blood and the venous blood from the lower extremity and the kidneys up to the heart. From here, this blood enters the right atrium and then the left atrium. After that, the blood passes into the left ventricle. The left and right sides of the fetal heart work together by using extra- and intra-cardiac shunts to fill the aorta for systemic circulation. The distal aorta finally terminates into two common iliac arteries which, in turn, branch off into internal and external iliac arteries. The umbilical arteries, which carry the blood back to the placenta, originate from the internal iliac arteries.

Conversely, in TRAP sequence, the deoxygenated blood of the pump twin does not reach the placenta completely. Part of it instead enters arterio-arterial anastomoses and travels in retrograde direction into one or both umbilical arteries, as well as into the systemic circulation of the acardiac twin. Thus, the “Twin Reversed Arterial Perfusion sequence” is created. The exact pathogenesis of TRAP sequence is still unknown, but it is a fact that the pump twin provides circulatory support to the acardiac twin from as early as the first trimester. However, the mere presence of vascular anastomoses in the placenta alone cannot lead to the development of TRAP sequence. Moreover, the presence of retrograde blood flow through arterio-arterial anastomoses increases the probability of TRAP sequence diagnosis in a suspected monochorionic twin pregnancy. Here, we are presenting a case of pregnancy with an undiagnosed TRAP sequence.

## Case presentation

A 26-year-old patient gravida 3 para 2 (G3P2+0) with two previous normal deliveries, was referred by a primary health center to our emergency labor room at (assumed) 37 weeks of pregnancy with complaints of vaginal leakage and a non-reassuring fetal status (less fetal movement and fetal bradycardia). The patient conceived during lactational amenorrhea and was unsure about her period of gestation. She attended only one antenatal check-up at a local health center, where she complained of excessive nausea and was diagnosed as being between 14 and 16 weeks pregnant. The patient was administered one shot of tetanus toxoid and prescribed iron, calcium, and folic acid tablets, along with the antiemetic treatment. Her previous obstetric history was uneventful, and all deliveries were institutional. Her last child was one year old at the time of her visit to the emergency labor room.

On general examination, she appeared dehydrated, dyspneic, and pale, and bipedal edema was present. Her blood pressure was 116/74 mmHg, and her pulse rate was 112 beats per minute. Upon obstetrical examination, her uterus was unduly enlarged, which suggested polyhydramnios, and the fetal heart sound was muffled. Doppler ultrasound measured the fetal heart rate at 102 beats per minute. On pelvic examination, leaking was observed per speculum. Upon vaginal examination, os was 3 to 4 cm dilated, and the cervix was in an anterior position and was 30% effaced. Furthermore, the membrane was absent, and the presenting part felt irregular and soft.

**Figure 1:**
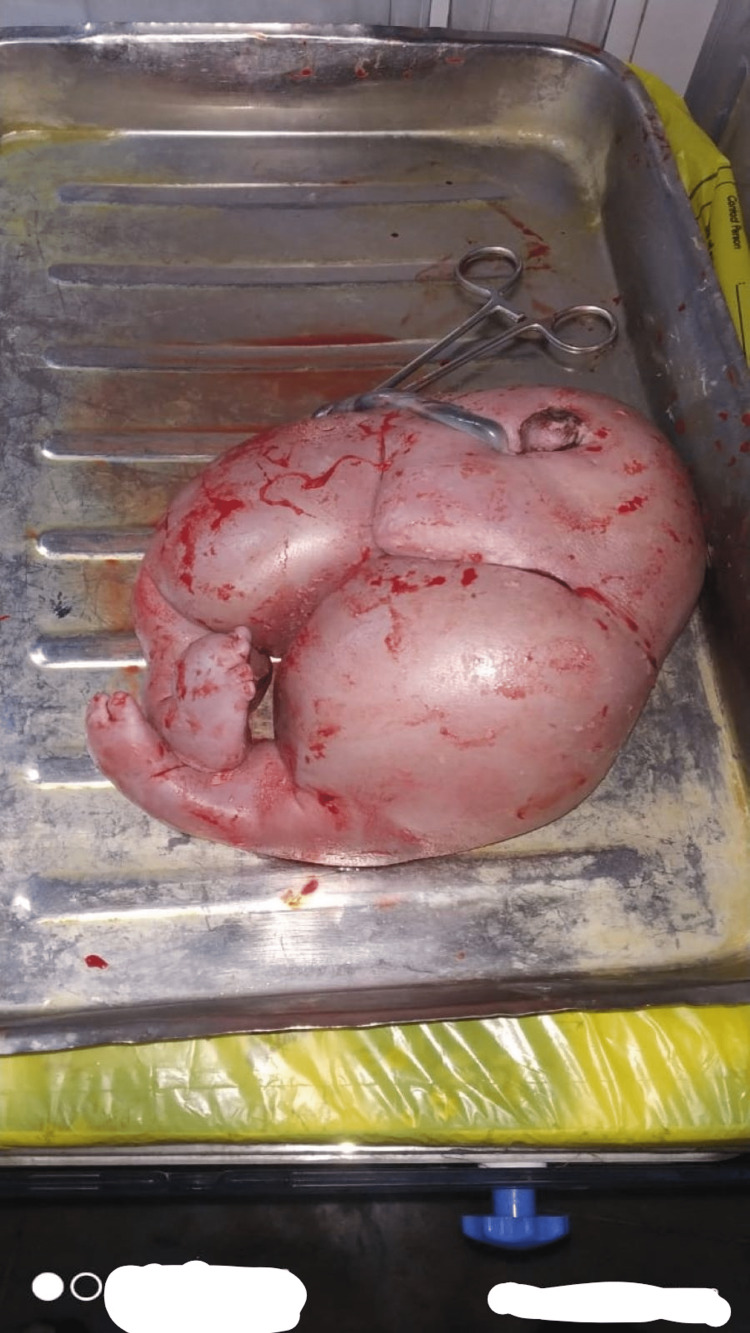
Acardiac Twin

Along with the treatment of the patient’s dehydration and intermittent oxygen inhalation, she was immediately transferred to the Operation Theatre, and an emergency lower segment cesarean section was performed. At the time of delivery, she was diagnosed as having monochorionic diamniotic twins with TRAP sequence as one of the twins was acardiac while the other was a living male baby (the pumping twin) with massive ascites, weight 2200 grams, and APGAR score at birth 3. The baby was immediately transferred to Special Newborn Care Unit (SNCU) where it was diagnosed with high-output heart failure. It did not survive longer than six hours post-delivery. Figure 1 shows the acardiac twin. The mother received grief counseling and milk suppression treatment. Her postpartum period was uneventful.

## Discussion

It is said that TRAP sequence is found mainly in monochorionic twins, but very rarely has it been reported in monochorionic triamniotic gestations [2]. Prenatally, TRAP sequence can be diagnosed using ultrasonography if there are characteristic findings of monochorionic multiple pregnancies. Such pathognomonic findings could be the fetuses having an absent cardiac activity or a rudimentary heart and retrograde arterial flow to the acardiac fetus, observed using a Doppler ultrasound test. TRAP sequence can be diagnosed during the very early stages of pregnancy [3], which was missed in our case due to the patient’s lack of awareness.

The presence of hydrops or high-output heart failure in the pump twin increases its mortality risk. These circumstances were present in our case study, leading to the pump twin’s demise. Other poor prognostic factors include abnormal Doppler velocimetry indices of the pump twin and the ratio of the weight of the acardiac twin to the weight of the pump twin > 0.7 [4-7].

Patients with a diagnosed TRAP sequence pregnancy should be referred to fetal medicine centers. Termination of such pregnancies should also be duly considered. The risk of genetic abnormalities seems to increase in TRAP sequence pregnancies [8, 9]. Hence, the recommendation for fetal genetic testing should be made to the patients. For patients interested in continuing with the pregnancy, conservative management or targeted cord occlusion of the acardiac twin can be advised. The procedure for cord occlusion requires high expertise and specialized equipment, which is offered by a limited number of institutions. Radiofrequency ablation and bipolar cord coagulation are contemporary modalities for cord occlusion therapy in TRAP sequence management.

## Conclusions

TRAP sequence pregnancies with poor prognostic factors require twin cord occlusion therapy or another form of intervention. Advancements in the field of fetal medicine have certainly improved the survival rate of such conditions. Termination of such pregnancies is also a valid option as it saves the patient from unnecessary obstetrical complications and morbidity. To avail of any of these options, a patient needs to be in regular antenatal care as ignoring its importance can be harmful to both mother and baby.
